# *FGD5-AS1* Is a Hub lncRNA ceRNA in Hearts With Tetralogy of Fallot Which Regulates Congenital Heart Disease Genes Transcriptionally and Epigenetically

**DOI:** 10.3389/fcell.2021.630634

**Published:** 2021-05-11

**Authors:** Xingyu Zhang, Yunqian Gao, Xiaoping Zhang, Xiaoqing Zhang, Ying Xiang, Qihua Fu, Bo Wang, Zhuoming Xu

**Affiliations:** ^1^Pediatric Translational Medicine Institute, Shanghai Children’s Medical Center, Shanghai Jiao Tong University School of Medicine, Shanghai, China; ^2^Department of Laboratory Medicine, Shanghai Children’s Medical Center, Shanghai Jiao Tong University School of Medicine, Shanghai, China; ^3^Faculty of Medical Science, Shanghai Jiao Tong University School of Medicine, Shanghai, China; ^4^Cardiac Intensive Care Unit, Department of Thoracic and Cardiovascular Surgery, Shanghai Children’s Medical Center, Shanghai Jiao Tong University School of Medicine, Shanghai, China

**Keywords:** congenital heart disease, gene expression, lncRNA, *FGD5-AS1*, tetralogy of Fallot

## Abstract

Heart development requires robust gene regulation, and the related disruption could lead to congenital heart disease (CHD). To gain insights into the regulation of gene expression in CHD, we obtained the expression profiles of long non-coding RNAs (lncRNAs) and messenger RNAs (mRNAs) in 22 heart tissue samples with tetralogy of Fallot (TOF) through strand-specific transcriptomic analysis. Using a causal inference framework based on the expression correlations and validated microRNA (miRNA)–lncRNA–mRNA evidences, we constructed the competing endogenous RNA (ceRNA)-mediated network driven by lncRNAs. Four lncRNAs (*FGD5-AS1*, *lnc-GNB4-1*, *lnc-PDK3-1*, and *lnc-SAMD5-1*) were identified as hub lncRNAs in the network. *FGD5-AS1* was selected for further study since all its targets were CHD-related genes (*NRAS*, *PTEN*, and *SMAD4*). Both *FGD5-AS1* and *SMAD4* could bind with hsa-miR-421, which has been validated using dual-luciferase reporter assays. Knockdown of *FGD5-AS1* not only significantly reduced *PTEN* and *SMAD4* expression in HEK 293 and the fetal heart cell line (CCC-HEH-2) but also increased the transcription of its interacted miRNAs in a cell-specific way. Besides ceRNA mechanism, RNAseq and ATACseq results showed that *FGD5-AS1* might play repression roles in heart development by transcriptionally regulating CHD-related genes. In conclusion, we identified a ceRNA network driven by lncRNAs in heart tissues of TOF patients. Furthermore, we proved that *FGD5-AS1*, one hub lncRNA in the TOF heart ceRNA network, regulates multiple genes transcriptionally and epigenetically.

## Introduction

Congenital heart disease/defect (CHD) is a group of structural abnormalities of the heart and great vessels that originated from embryonic development. It is the most common birth defect with an incidence of ∼1% (van der Linde et al., 2011). Although most CHD patients could survive with surgical repair, they would be confronted with a high risk of multiple symptoms such as cardiac arrhythmias and heart failure (Egbe et al., 2014). To make better strategies for the prevention or therapy for CHD, identification of the predisposing factors that cause abnormal fetal heart development is always a prerequisite. Epidemiological studies have suggested that genetic or environmental factors could be merely identified in 20–30% of CHD cases. Genetic factors such as single-gene disorders, gross chromosomal anomalies/aneuploidies, and pathogenic copy number variations were, respectively, found in 3–5%, 8–10%, and 3–25% of CHD cases (Cowan and Ware, 2015). Based on the genetic sequence variations in patients, identifying disease-associated genes is the most practical strategy for interpreting the genetic factors of CHD.

Apart from another 2% of cases attributed to environmental factors, the remaining unexplained CHD cases are presumed to be multifactorial (oligogenic, polygenic, or a combination of genetic and environmental factors) (Cowan and Ware, 2015). Therefore, epigenetic factors should be an important concern in CHD studies since they reflect the interactions between environmental and genetic factors. Actually, it has been revealed that epigenetic abnormalities contribute to the development of CHD, and related gene expression is altered in the patients’ hearts (Serra-Juhe et al., 2015; Grunert et al., 2016). Elucidating the mechanisms of regulation of gene expression should greatly help in understanding the etiology of CHD.

In the past, most efforts were exerted in investigating the roles of micro RNAs (miRNAs) and/or messenger RNAs (mRNAs) in CHD. Significant progress has been made focusing on the role of miRNAs in heart development (Das et al., 2020). It has also been revealed that long non-coding RNAs (lncRNAs), pseudogenes, circular RNAs (circRNAs), and mRNAs compete for the same pool of miRNAs (Tay et al., 2014), which is termed the ceRNA (competing endogenous RNA) mechanism. Although lncRNAs have received much attention for their regulatory role in gene expression and potential value in diagnosis and treatment recently, their regulatory functions have not been characterized systematically in CHD samples. To gain a better understanding of the gene expression profiles in the heart of CHD, we performed strand-specific RNA sequencing (RNAseq) on myocardial samples of the right heart ventricle tissues from 22 children with tetralogy of Fallot (TOF), a severe form of CHD. By constructing lncRNA-driven miRNA regulatory networks, we identified four hub lncRNAs (*FGD5-AS1*, *lnc-GNB4-1*, *lnc-PDK3-1*, and *lnc-SAMD5-1*) that regulate mRNA expression through miRNAs and also investigated the molecular basis of *FGD5-AS1*.

## Materials and Methods

### Ethical Approval

The Ethics Committee of the Shanghai Children’s Medical Center reviewed and approved this study (SCMCIRB-K2017009). All procedures performed in this study involving human participants were in accordance with the ethical standards of the institutional and/or national research committee as well as the 1964 Helsinki declaration, including its later amendments or comparable ethical standards. Informed consents were obtained from all individual participants’ parents in the study.

### Tissue Samples

Right heart ventricle tissues were collected from 22 patients with main cardiac malformation of TOF requiring surgical reconstruction. At surgery, the diagnosis and anatomy of TOF were confirmed and ventricular myocardial tissues were retrieved. Samples were immediately stored in RNALater (Ambion, Austin, TX, United States) at −80°C for subsequent processing.

### Cell Culture

The human embryonic kidney (HEK) 293 cell line as well as the CCC-HEH-2 human cardiac myocytes were purchased from the Type Culture Collection of the Chinese Academy of Sciences, Shanghai, China. Cells were cultured in Dulbecco’s modified Eagle’s medium (DMEM) (Gibco, Thermo Fisher Scientific, Inc., Waltham, MA, United States) containing 10% fetal bovine serum (FBS) (Gibco) and 0.1% penicillin/streptomycin (NCM Biotech, Suzhou, Jiangsu, China) at 37°C with 5% CO_2_.

### Cell Infection and Transfection

The specific short hairpin RNAs (shRNAs) against *FGD5-AS1* [shRNA#1, #2, #3, and #mix (mixture of shRNA#1, #2, and #3)] and the corresponding negative control, along with the green fluorescent protein (GFP)-expressing lentiviral vector and a puromycin resistance marker, were synthesized by GenePharma (Shanghai, China). For virus production, the shRNA plasmids psPAX2 and pMD2.G were concurrently transfected into HEK 293 cells by Lipofectamine 2000 (Invitrogen, United States). Then, the HEK 293 as well as the CCC-HEH-2 cell lines were infected with lentivirus and followed puromycin treatment for several days in order to acquire the stable knockdown (KD) cell lines.

The hsa-miR-107, hsa-miR-320a-3p, hsa-miR-454-3p, and hsa-miR-421 mimics and the negative control (NC) mimics were obtained from GenePharma. miR mimics were transfected into HEK 293 cells by Lipofectamine 2000 (Invitrogen, United States). The sequences for the shRNA and miR mimics are shown in Supplementary Table 1.

### RNAseq Analysis

Tissues or cell samples were lysed in TRIzol (Ambion, Austin, TX, United States). Total RNA was extracted using the miRNeasy kit (Qiagen, Hilden, Germany) according to the manufacturer’s instructions.

A total amount of 3 μg RNA per sample was used as the input material for the RNA sample preparations. Firstly, ribosomal RNA (rRNA) was removed using Epicentre Ribo-zero^TM^ Kit (Illumina Inc., San Diego, CA, United States), and rRNA-free residue was cleaned up by ethanol precipitation. Subsequently, sequencing libraries were generated using the rRNA-depleted RNA by the NEBNext Ultra TM Directional RNA Library Prep Kit for Illumina (NEB, Beverly, MA, United States) following the manufacturer’s recommendations. In order to select complementary DNA (cDNA) fragments of preferentially 250–300 bp in length, the library fragments were purified with the AMPure XP system (Beckman Coulter, Beverly, MA, United States). Then, 3 μl USER Enzyme (NEB, Beverly, MA, United States) was used with the size-selected, adaptor-ligated cDNA at 37°C for 15 min followed by 5 min at 95°C before PCR. PCR was performed with Phusion high-fidelity DNA polymerase, universal PCR primers, and index (x) primer. The PCR products were purified (AMPure XP system) and library quality was assessed on the Agilent Bioanalyzer 2100 system. The clustering of the index-coded samples was performed on a cBot Cluster Generation System using the HiSeq 4000 PE Cluster Kit (Illumina Inc., San Diego, CA, United States) according to the manufacturer’s instructions. After cluster generation, the library preparations were sequenced on the Illumina HiSeq 4000 platform and 150-bp paired-end reads were generated.

Read pairs with low quality (e.g., proportion of bases with sQ ≤ 5 greater than 50%, proportion of *N* greater than >10%, and 5′ adaptor contamination) were removed. The remaining clean data were aligned to human reference genome (version GRCh38) using STAR (Dobin et al., 2013) with Encyclopedia of DNA Elements (ENCODE)^1^ standard options for long RNAseq pipeline. Gene-level read counts were estimated using RNA-seq by Expectation Maximization (RSEM) software (Li and Dewey, 2011). Differential gene expression analysis was performed using the DESeq2 (Love et al., 2014) package. Genes with fold change greater than 2 and adjusted *p*-value lower than 0.05 were defined as differentially expressed genes. Functional enrichment analysis was performed with the clusterProfiler (Yu et al., 2012) package.

Additionally, we used another dataset containing the RNAseq data of 22 TOF heart samples (PRJNA156781) to validate the co-expression relationship of hub lncRNAs and their mRNA which we identified. The data processing procedures were the same as described above.

### Construction of lncRNA-Driven ceRNA Gene Expression Network

A method for the identification of the lncRNA-related miRNA sponge regulatory network (Zhang et al., 2018) was implemented by analyzing our transcriptomic data of heart tissues. Generally, the method includes the following procedures:

(a) Putative miRNA–target (miRNA–mRNA and miRNA–lncRNA) interactions were integrated from several experimentally validated miRNA–target interaction databases such as miRTarBase v7.0 (Chou et al., 2018), TarBase v7.0 (Vlachos et al., 2015), NPInter v3.0 (Hao et al., 2016), and LncBase v2.0 (Paraskevopoulou et al., 2016). In total, 9,318 and 173,468 unique putative interactions were, respectively, collected for miRNA–mRNA and miRNA–lncRNA.
(b) Identify lncRNA–mRNA pairs that have a significant sharing of miRNAs based on the miRNA–target interactions.
(c) Extract matched lncRNA and mRNA expressions from the TOF heart transcriptomic data for the previously identified lncRNA–mRNA pairs and calculate the causal effects using the parallel IDA algorithm (Le et al., 2019).
(d) Construct the lncRNA-mediated miRNA regulatory network by taking advantage of the *corPvalueFisher* method of the WGCNA package (Langfelder and Horvath, 2008). The lncRNA–mRNA interaction with adjusted *p* < 0.05 (BH) were considered as sponge lncRNA–mRNA regulatory relationships.

### ATACseq Analysis

Approximately 5 × 10^4^ fresh CCC-HEH-2 cells, each with sh-*FGD5-AS1* for knockdown or the corresponding negative control (described in section “Cell Infection and Transfection”) were collected by centrifugation at 500 × *g* and washed twice with cold phosphate-buffered saline (PBS). Nuclei-enriched fractions were extracted with cold resuspension buffer (0.1% NP-40, 0.1% Tween 20, and 0.01% Digitonin) and washed out with 1 ml of cold resuspension buffer containing 0.1% Tween 20 only. Nuclei pellets were collected by centrifugation and resuspended with transposition reaction buffer containing Tn5 transposases (Nextera XT Library Kit, cat. no. FC-131-1096, Illumina). Transposition reactions were incubated at 37°C for ∼30 min, followed by DNA purification using the DNA Cleanup and Concentration Kit (cat. no. D4013, ZYMO Research). Libraries were amplified with Nextera barcodes and high-fidelity polymerase (cat. no. M0541, New England Labs) and purified using Agencourt AMPure XP beads (cat. no. A63880, Beckman Coulter). Libraries were sequenced on HiSeq 4000 for 150-bp paired-end sequencing. Raw fastq files were fed to nf-core (Ewels et al., 2020) ATACseq (assay for transposase-accessible chromatin with sequencing) pipeline using GRCh38.

### RT-qPCR

The total cellular RNA was extracted with TRIzol reagent (Ambion, Austin, TX, United States) and reverse transcribed into cDNA with PrimeScript RT Reagent Kit (Takara, Dalian, China). Quantitative reverse transcription PCR (RT-qPCR) was conducted with TB Green Premix Ex Taq II Kit (Takara, Dalian, China) under the CFX 9600 Real-Time PCR detection system (Bio-Rad Laboratories, Inc., Hercules, CA, United States). Based on the 2^−ΔΔCt^ method, fold changes of the target genes, as well as an expression quantitative trait loci (eQTL) gene (*RBSN*), of *FGD5-AS1* were calculated with *GAPDH* or U6 as the internal control. Primers for PCR are shown in Supplementary Table 1. For qPCR validation in TOF heart tissue samples, gene expression was reported as 2^−ΔCt^ relative to *GAPDH*.

### Luciferase Reporter Assay

The plasmids used for the luciferase reporter assay were constructed with pmirGLO dual-luciferase vector (Promega, Madison, WI, United States), including *FGD5-AS1* WT/MUT (wild type/mutation), *PTEN* WT/MUT, and *SMAD4* WT/MUT. The *FGD5-AS1*, *PTEN*, and *SMAD4* MUT vectors were constructed within the mutated seed region of the corresponding miRNAs. These plasmids as well as the pmirGLO vector were, respectively, transfected with the hsa-miR-107 mimics/hsa-miR-320a-3p mimics/hsa-miR-454-3p mimics/hsa-miR-421 mimics or the NC mimics into the HEK 293 cells for 48 h. Lastly, luciferase activities were detected using the Dual-Luciferase Reporter Assay System (Promega, Madison, WI, United States). Information on the construction of plasmids is shown in Supplementary Table 1.

### Cell Proliferation and Apoptosis Assay

Cell proliferation assay was detected using TransDetect EdU Flow Cytometry Kit-647 Fluorophore (TransGen Biotech, Beijing, China). The cells were cultured with 5-ethynyl-2′-deoxyuridine (EdU) solution for 2 h and then fixed by 1 × EdU permeabilization buffer. Subsequently, the assay was visualized by the FACScan System (Bio-Rad Laboratories, Inc., Hercules, CA, United States).

The apoptotic assay was performed with the APC Annexin V Apoptosis Detection Kit with 7-AAD (BioLegend Inc., San Diego, CA, United States). The cells were washed with staining buffer and resuspended in annexin V binding buffer, then incubated with APC Annexin V and 7-AAD for 15 min at room temperature. The percentages of apoptotic cells were assessed by the FACScan System (Bio-Rad Laboratories, Inc., Hercules, CA, United States) according to the manufacturer’s guide.

### Western Blotting

Western blotting was performed to assess the expression of SMAD4 in infected HEK 293 and CCC-HEH-2 cells. Total cells were disintegrated using lysis buffer (Beyotime Biotechnology, Shanghai, China), then the extracted proteins were separated with 10% SDS-PAGE (Beyotime, Nantong, Jiangsu, China) and transferred onto PVDF membranes (Roche Diagnostic Corporation, Indianapolis, IN, United States). The membranes were blocked by 5% non-fat milk for 2 h and cultured with primary antibodies of SMAD4 (1:1,000; cat. no. 38454, Cell Signaling Technology) and GADPH (1:4,000; cat. no. 2118, Cell Signaling Technology). Sequentially, the membranes were incubated with secondary antibodies (1:10,000; cat. no. 7074, Cell Signaling Technology). Lastly, proteins were visualized using enhanced chemiluminescence (ECL) detection kits (Millipore Corp., Millipore, Billerica, MA, United States). The quantification of the results was analyzed by ImageJ.

### Statistical Analysis

SPSS 16.0 software was used for statistical analysis. Differences between two groups were analyzed by implementing Student’s *t*-test, and *p* < 0.05 was regarded as statistically significant. All data were expressed as mean ± SD.

## Results

### Construction of lncRNA-Driven ceRNA Regulatory Network

**FIGURE 1:**
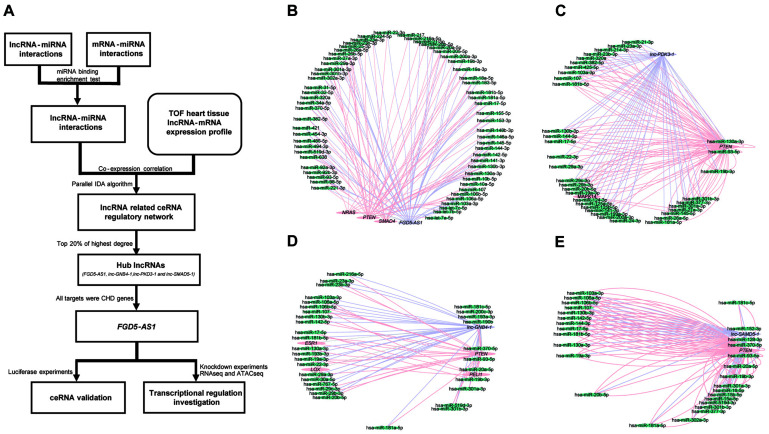
Workflow of the entire study and the competing endogenous RNA (ceRNA) regulatory networks of each hub long non-coding RNA (lncRNA) in tetralogy of Fallot (TOF) hearts. **(A)** The workflow for the entire study. **(B–E)** The four networks all contain three types of genes, namely lncRNAs (*purple*), mRNAs (*pink*), and miRNAs (*green*). The *edges connected between nodes* indicate their co-expression relationship. The networks of the four hub lncRNAs (*FGD5-AS1*, *lnc-GNB4-1*, *lnc-PDK3-1*, and *lnc-SAMD5-1*) are presented, respectively.

Clinical information of the TOF samples has been listed in Supplementary Table 2. The detailed workflow of the entire study is presented in Figure 1A. lncRNAs/mRNAs with low expressions (total counts of 22 samples lower than 100) were removed, then the remaining 15,036 mRNAs and 14,377 lncRNAs were kept for further analysis. In total, 36 lncRNA/mRNA-related miRNA sponge interactions involving 24 lncRNAs and 23 mRNAs were identified. Since it has been suggested that nearly 20% of the nodes in a biological network tend to be essential (Song and Singh, 2013), we selected the top 20% of lncRNAs with the highest degrees (the number of edges connected with a node) as the hub lncRNAs: *FGD5-AS1*, *lnc-GNB4-1*, *lnc-PDK3-1*, and *lnc-SAMD5-1* (Figure 1, Supplementary Figure 1, and Supplementary Table 3).

### LncRNA–miRNA–mRNA Relationship Validation

**FIGURE 2:**
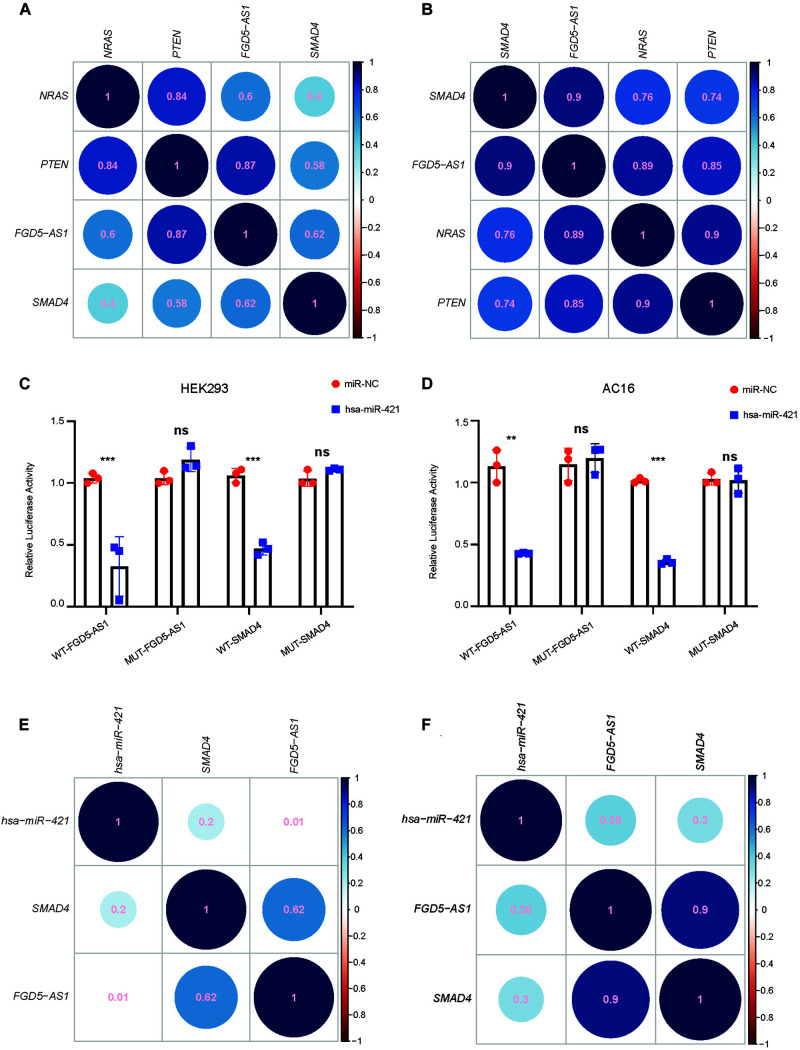
*FGD5-AS1* competing endogenous RNA (ceRNA) mechanism validation. The correlation coefficients between *FGD5-AS1* and its ceRNA targets for our dataset **(A)** and the GEO: PRJNA156781 dataset **(B)** are shown. For lncRNA *FGD5-AS1* and mRNA *SMAD4*, hsa-miR-421 was validated in the HEK 293 **(C)** and AC16 **(D)** cell lines, respectively. The correlation coefficients between hsa-miRNA-421 and *FGD5-AS1*/*SMAD4* in our dataset **(E)** and the GEO: PRJNA156781 dataset **(F)** are also shown. n.s., not significant; The number of asterisks indicated the corresponding statistical significance (*p-value*). *p < 0.05; **p < 0.01; ***p < 0.001.

We selected *FGD5-AS1* for experimental validation since all its target mRNAs (*SMAD4*, *PTEN*, and *NRAS*) were known as CHD genes. According to our dataset, the expressions of *FGD5-AS1* and its three target mRNAs (Pearson’s correlation coefficients: *NRAS*, 0.60; *PTEN*, 0.87, and *SMAD4*, 0.62) were highly correlated (Figure 2A). In order to validated such co-regulation relationship, we analyzed another dataset of TOF heart tissue gene expressions (Grunert et al., 2016). The validation was in accordance with our results (Figure 2B): the Pearson’s correlation coefficients were 0.9, 0.85, and 0.89 for *SMAD4*, *PTEN*, and *NRAS*, respectively. The expressions of *FGD5-AS1*, *NRAS*, *PTEN*, *SMAD4*, hsa-miR-107, hsa-miR-320a-3p, hsa-miR421, and hsa-miR-454-3p were also validated in 12 additional TOF heart tissues using RT-qPCR. The three mRNAs showed high expression correlations with *FGD5-AS1* (Pearson’s correlation coefficients: *NRAS*, 0.73; *PTEN*, 0.94; and *SMAD4*, 0.71) (Supplementary Figure 2). *FGD5-AS1* shared 48 miRNAs (hypergeometric enrichment test: *p* = 0) with *PTEN*, 21 miRNAs (hypergeometric enrichment test: *p* = 4.75 × 10^–9^) with *SMAD4*, and nine miRNAs (hypergeometric enrichment test: *p* = 7.26 × 10^–6^) with *NRAS*. Besides *FGD5-AS1*, *PTEN* shared multiple miRNAs with four other hub lncRNAs (The number of shared miRNAs: *lnc-IL17B-2*, 46; *lnc-GNB4-1*, 30; *lnc-PDK3-1*, 29; *lnc-SAMD5-1*, 25). *SMAD4* shared 22 miRNAs with *GAS5* and seven miRNAs with *lnc-ZCCHC7-2*. *NRAS* shared only four miRNAs with *lnc-ZNF124-1* other than *FGD5-AS1*. Accordingly, *PTEN* and *SMAD4* should play more important roles in the ceRNA network. We selected miRNAs for each of *SMAD4* (hsa-miR-421 and hsa-miR-454-3p) and *PTEN* (hsa-miR-107 and hsa-miR-320a-3p) for binding validation since the four miRNAs have been reported to have important association with heart development or congenital heart disease (O’Brien et al., 2012; Zhou et al., 2014; Colpaert and Calore, 2019; Grunert et al., 2019). The predicted lncRNA–miRNA–mRNA interactions were validated in the HEK 293 cell line and a cardiac cell line (AC16). A previous reported mRNA–miRNA target pair (*CRYAB* and hsa-miR-491) (Wang et al., 2017) was used as the positive control for all luciferase assays (Supplementary Figure 3). Our results suggested that only hsa-miR-421 could combine with both *FGD5-AS1* and *SMAD4* in the HEK 293 and AC16 cell lines (Figures 2C,D). Meanwhile, the validated axis of *FGD5-AS1*/hsa-miR-421/*SMAD4* has also been analyzed at the co-expression level with datasets of TOF (Figures 2E,F; Grunert et al., 2016, 2019).

### Effect of *FGD5-AS1* KD on Gene Expression, Cell Proliferation, and Apoptosis

**FIGURE 3:**
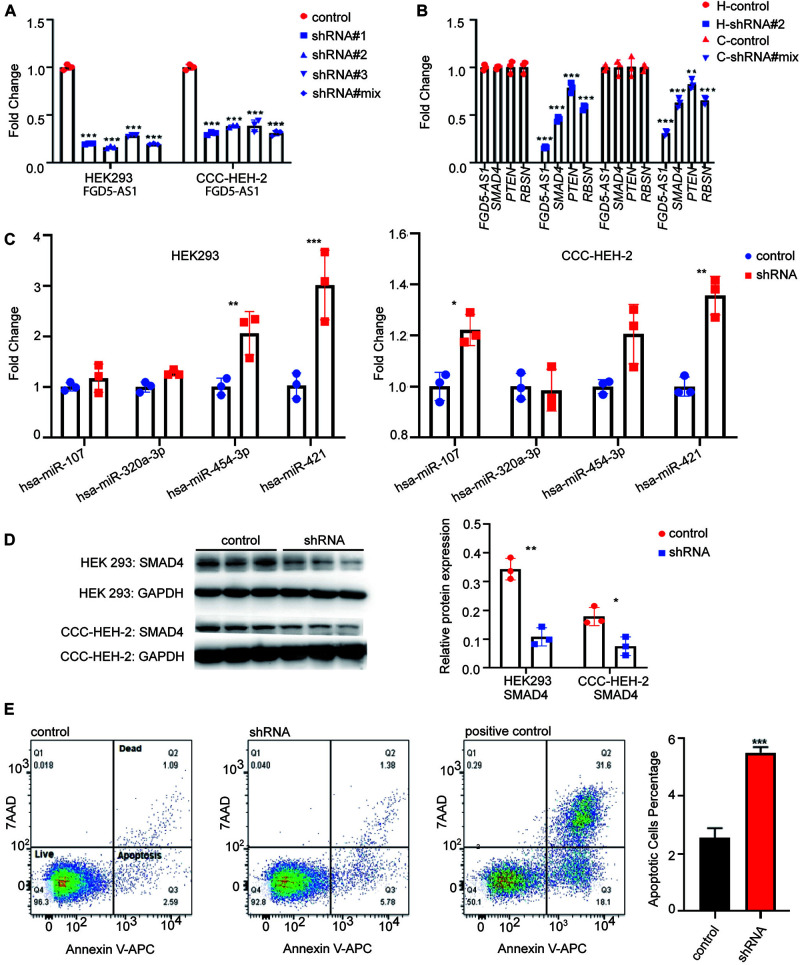
lncRNA *FGD5-AS1* suppressed the apoptosis. **(A)** Quantitative reverse transcription PCR (RT-qPCR) assays were performed to detect the interference efficiency of *FGD5-AS1* in the HEK 293 and CCC-HEH-2 cell lines. **(B)** RT-qPCR assays suggested a decreased transcriptional level of mRNA (*SMAD4*, *PTEN*, and *RBSN*) in two knockdowns (KD) of *FGD5-AS1* cell lines (*H*, HEK 293 cell line; *C*, CCC-HEH-2 cell line). **(C)** RT-qPCR assays of four miRs for previous validation in the *FGD5-AS1* KD HEK 293 and CCC-HEH-2 cell lines. **(D)** Western blotting was used to verify that interfered *FGD5-AS1* can affect the expression of SMAD4, and the relative protein expression was analyzed. **(E)** Flow cytometry was performed to detect apoptosis in the *FGD5-AS1* KD CCC-HEH-2 cell line. The positive controls were CCC-HEH-2 cells treated with apoptosis induction drugs. n.s., not significant; The number of asterisks indicated the corresponding statistical significance (*p*-value). *p < 0.05; **p < 0.01; ***p < 0.001.

We synthesized three shRNA vectors and its corresponding negative control for KD experiments of *FGD5-AS1*. shRNA#2 and shRNA#mix had the best performance in the HEK 293 and CCC-HEH-2 cell lines, respectively (Figure 3A). KD of *FGD5-AS1* could significantly decrease the transcriptional expressions of *SMAD4* and *PTEN*. Additionally, based on the records of FANTOM5, *RNSB* had an eQTL relationship with *FGD5-AS1*, which was also validated by our KD experiments (Figure 3B). Interestingly, the *FGD5-AS1* KD HEK 293 cell line prepared with shRNA#2 showed elevated expressions of both hsa-miR-454-3p and hsa-miR-421 (Figure 3C). hsa-miR-107 and hsa-miR-421 were significantly upregulated in the *FGD5-AS1* KD CCC-HEH-2 cell line. When *FGD5-AS1* was suppressed, SMAD4 were also downregulated at the protein level (Figure 3D).

We also evaluated the cell proliferation and apoptosis in *FGD5-AS1* KD CCC-HEH-2 cells. The results indicated that the apoptosis was significantly elevated in *FGD5-AS1* KD cells (Figure 3E). However, the proliferation showed no statistical significance (Supplementary Figure 4).

### Molecular Basis of *FGD5-AS1* Regulation Revealed by RNAseq and ATACseq

**FIGURE 4:**
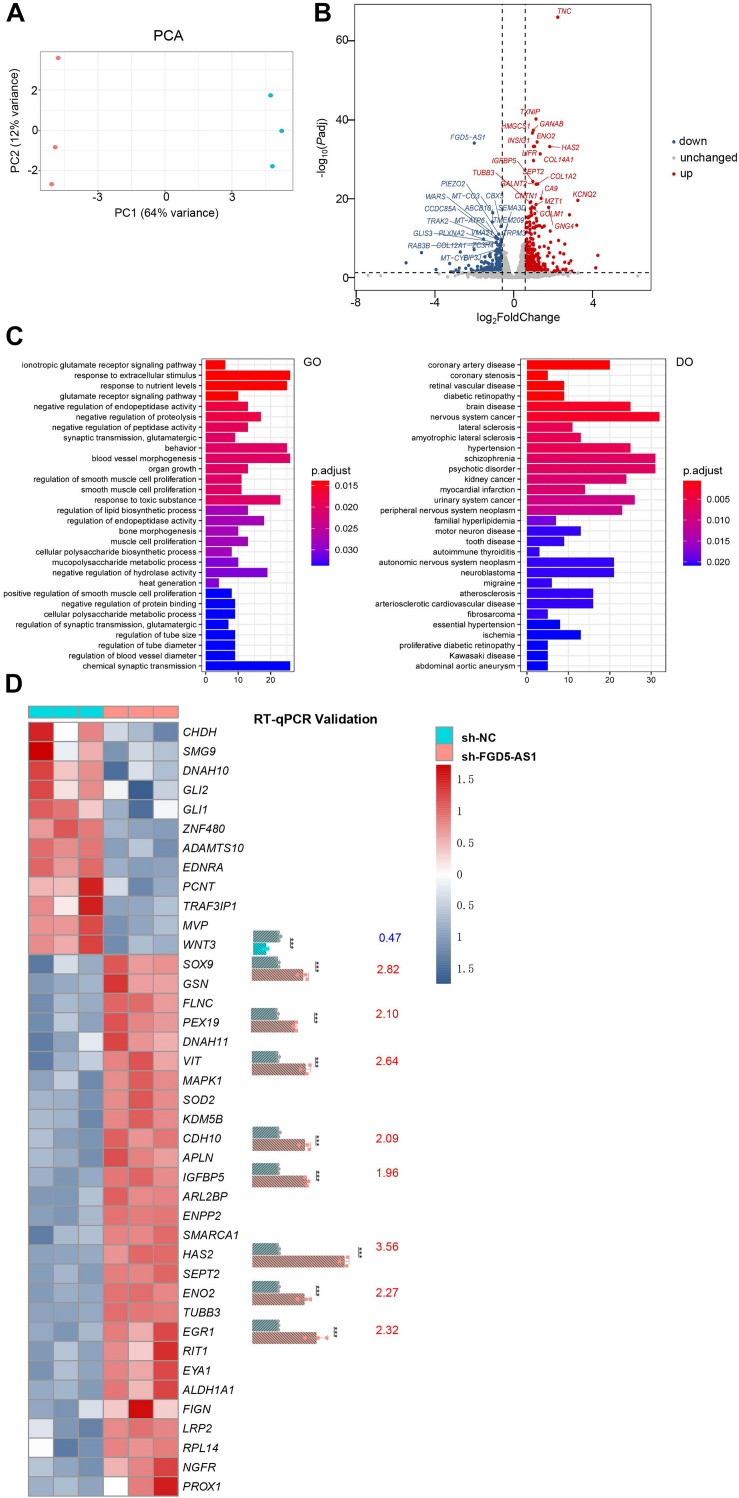
RNA sequencing (RNAseq) analysis of *FGD5-AS1* knockdown (KD) CCC-HEH-2 cells. **(A)** Principal component analysis (PCA) plot of RNAseq. Control and shRNA KD CCC-HEH-2 cell samples are shown in *red* and *cyan*, respectively. **(B)** Volcano plot of differential gene expression analysis between the control and the *FGD5-AS1* KD CCC-HEH-2 cell line. Symbols of the top 20 significantly up/downregulated genes are labeled. **(C)** Gene Ontology (GO) and Disease Ontology (DO) enrichment analysis of the upregulated genes in RNAseq. **(D)** Heatmap of the 41 congenital heart disease (CHD)-related genes within differential expression in RNAseq. Nine of them were validated by RT-qPCR (*WNT3*, *SOX9*, *PEX19*, *VIT*, *CDH11*, *IGFBP5*, *HAS2*, *ENO2*, and *EGR1*), and the fold change values are labeled. n.s., not significant; The number of asterisks indicated the corresponding statistical significance (*p-value*). *p < 0.05; **p < 0.01; ***p < 0.001.

**FIGURE 5:**
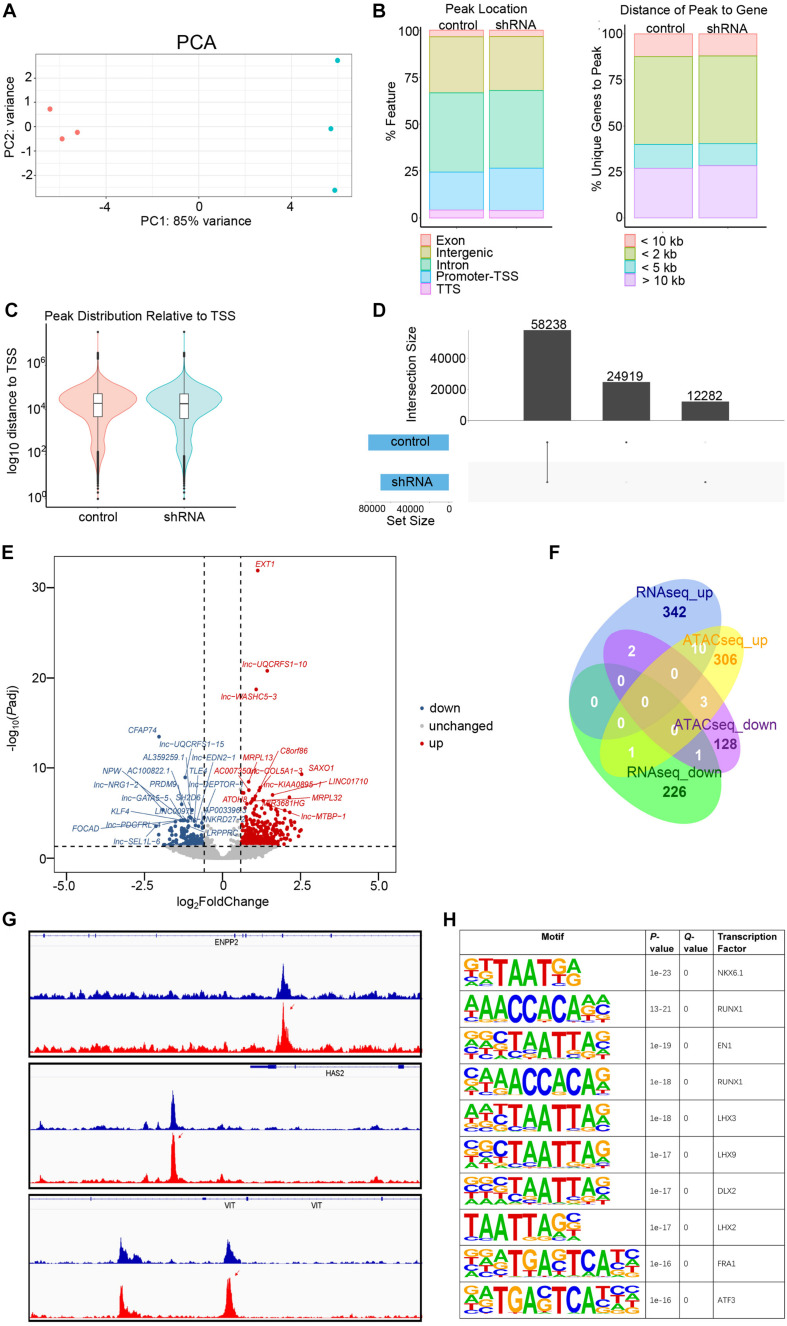
Assay for transposase-accessible chromatin with sequencing (ATACseq) analysis of *FGD5-AS1* knockdown (KD) CCC-HEH-2 cells. **(A)** Principal component analysis (PCA) plot of ATACseq. Control and shRNA KD CCC-HEH-2 cell samples are shown in *red* and *cyan*, respectively. **(B)** Peak location comparison of the gene features. **(C)** Distance of peaks relative to the closest gene. **(D)** Peak distribution relative to the transcriptional start site. **(D)** Consensus peak number comparison of control of the KD samples. **(E)** Volcano plot of differential accessible region analysis. The top 20 significantly up/downregulated regions are labeled as the closest gene symbol. **(F)** Venn plot of differential gene expression analysis and differential accessible region analysis based on gene annotation. **(G)** Peak comparison of the differential accessible regions identified in *ENPP2*, *HAS2*, and *VIT*. **(H)** Enriched known motifs of the transcription factors within the differential accessible region sequences. Only the top 10 significant motifs are shown.

We performed RNAseq and ATACseq on *FGD5-AS1* KD CCC-HEH-2 cells to reveal the molecular basis of *FGD5-AS1* regulation (Figures 4, 5). KD of *FGD5-AS1* caused substantial transcriptional changes in CCC-HEH-2 cells: 354 genes were upregulated and 228 genes were downregulated (Figure 4B and Supplementary Table 4). Functional enrichment analysis revealed that only the upregulated genes were enriched in Gene Ontology (GO) terms such as ionotropic glutamate receptor signaling pathway, blood vessel morphogenesis, and organ growth. In the meantime, merely the upregulated genes were enriched in multiple Disease Ontology (DO) terms such as coronary artery disease, coronary stenosis, hypertension, and myocardial infarction (Figure 4C and Supplementary Table 5). Forty-one of these genes with differential expressions were reported as CHD-related genes, and we selectively validated nine of them (*WNT3*, *SOX9*, *PEX19*, *VIT*, *CDH11*, *IGFBP5*, *HAS2*, *ENO2*, and *EGR1*) using RT-qPCR (Figure 4D).

In total, 58,238 consensus regions with peak signals were revealed by ATACseq in *FGD5-AS1* KD and control cells (Figure 5). Differential accessibility region analysis identified 517 regions (376 with increased accessibility and 141 with reduced accessibility) (Figure 5E and Supplementary Table 6). Ten regions with increased accessibility were involved in genes with significant upregulated expressions (Figure 5F), and three of them were known CHD genes: *ENPP2*, *HAS2*, and *VIT* (Figure 5G). A set of enriched known motifs of transcription factors within the DAR sequences were also identified (Figure 5H and Supplementary Table 7).

## Discussion

Recently, gene regulation of non-coding transcripts, especially miRNAs (Smith et al., 2015; Hoelscher et al., 2017; Tian et al., 2017) and lncRNAs (Scheuermann and Boyer, 2013; Sallam et al., 2018) in heart development as well as CHD, has been underlined. It has been suggested that non-coding RNAs have profound effects on gene regulation. Except for lncRNAs, the expression profiles of mRNAs and miRNAs have also been systematically explored in CHD heart tissues (Bittel et al., 2011; Grunert et al., 2016, 2019; Wang et al., 2018). In the present study, we performed strand-specific RNAseq on heart tissues from 22 patients with TOF to retrieve the gene expression profiles of mRNAs and lncRNAs. Comparable numbers of evidently expressed mRNAs (15,036) and lncRNAs (14,377) were identified in these heart tissues. We focused on elucidating the lncRNA-related ceRNA regulatory network in CHD heart tissues. Since expression correlation-based methods only reflect the indirect or competitive relationships between sponge lncRNAs and mRNAs, we implemented a causal inference method to identify the lncRNA-driven ceRNA gene expression networks for this research. Using their interactive relationship with miRNAs retrieved from public databases with validation, we identified four hub lncRNAs that should play important roles in gene expression regulation with ceRNA mechanism in heart tissues with TOF. All four hub lncRNAs showed obvious enrichment of the shared miRNAs with their target mRNAs. We selected *FGD5-AS1* for further validation since its target mRNAs *NRAS*, *PTEN*, and *SMAD4* were all known CHD genes. *FGD5-AS1* shared nine, 48, and 21 miRNA interactions with *NRAS*, *PTEN*, and *SMAD4*, respectively (Supplementary Table 3).

To our knowledge, the regulatory function of *FGD5-AS1* in heart development has not been reported. *FGD5-AS1* was first identified as being involved in the lncRNA-associated ceRNA network of periodontitis (Li et al., 2018), and the upregulation of *FGD5-AS1* could protect against periodontitis *via* regulating the miR-142-3p/*SOCS6*/*NF-*κ*B* signals (Chen et al., 2019). *FDG5-AS1* was then reported to protect oxygen–glucose deprivation and simulated reperfusion-induced neurons injury *via* acting as a ceRNA for miR-223 to mediate *IGF1R* expression (Zhang et al., 2019). Computational analysis also identified *FGD5-AS1* as a key lncRNA for acute myocardial infarction. Other evidence of the regulatory functions of *FGD5-AS1* came from studies of cancers such as colorectal cancer (Li et al., 2019), non-small lung cancer (Fan et al., 2020; Fu et al., 2020), gastric cancer (Gao et al., 2020a), melanoma (Gao et al., 2020b), oral cancer (Liu et al., 2020), glioblastoma (Su et al., 2020), glioma (Lin et al., 2020; Zhao et al., 2020), renal cell carcinoma (Yang et al., 2020), and hepatocellular carcinoma (Zhang and Lou, 2020). A variety of related miRNAs (e.g., miR-223, miR-140-5p, miR-383, miR-153, miR-103a-3p, miR-129-5p, and miR-5590-3p) were also reported as interacting with *FGD5-AS1* in the above-mentioned works. Therefore, *FGD5-AS1* should be a versatile and pleiotropic master regulator of gene expression as a sponge lncRNA for miRNAs. To validate the lncRNA–miRNA–mRNA regulatory relationship that we identified in heart tissues with TOF, we selected two miRNAs for each of the *FGD5-AS1* targets: *PTEN* (hsa-miR-107 and hsa-miR-320-3p) and *SMAD4* (hsa-miR-421 and hsa-miR-454-3p) for interaction validation. Only hsa-miR-421 was confirmed to interact with both its lncRNA (*FGD5-AS1*) and mRNA (*SMAD4*) partners in the HEK 293 and AC16 cell lines using the dual-luciferase reporter assays (Figures 2C,D). Since the miRNA–lncRNA and miRNA–mRNA interactions we retrieved from databases were experimentally verified, the possible explanation for the situation is the cell type-specific effect of miRNAs, which have been observed in a previous *PTEN* ceRNA analysis (Tay et al., 2011). We then performed KD experiments to validate the regulatory effect of *FGD5-AS1* on its target *PTEN* and *SMAD4*, as well as *RBSN*, a gene with expression quantitative trait loci relationship with *FGD5-AS1*. Our results showed that KD of *FGD5-AS1* could robustly decrease the transcriptional levels of its target mRNAs in the HEK 293 and CCC-HEH-2 cell lines. Moreover, miRNA regulations at the expression level also affect the identification of lncRNA-related miRNA sponge interactions (Zhang et al., 2018). We found that both the *SMAD4*-interacting miRNAs (hsa-miR-454-3p and hsa-miR-421) were upregulated in *FGD5-AS1* KD HEK 293, and hsa-miR-421 was also upregulated in CCC-HEH-2 cells, which supported that the regulatory role of *FGD5-AS1* should be cell-specific.

Previous studies have identified that hsa-miR-421 was significantly upregulated in the right ventricular myocardium than the normally developing myocardium (O’Brien et al., 2012). In this study, the negative correlation of hsa-miR-421 and *SMAD4* was firstly reported. Subsequent KD and overexpression experiments of hsa-miR-421 indicated its significant inverse correlation with *SOX4*, which is a key regulator of Notch signaling (Bittel et al., 2014). Our results provided the evidence that the *FGD5-AS1*/hsa-miR-421/*SMAD4* axis should be a key cardiac development regulator contributing to TOF. In future experiments, it will be of interest to reveal how *FGD5-AS1* represses hsa-miR-421 during heart development.

Since *FGD5-AS1* regulates its potential binding miRNAs, it should have other regulatory roles other than ceRNA mechanism. We then tried to reveal additional roles for *FGD5-AS1* by preparing the *FGD5-AS1* KD CCC-HEH-2 cell line. Based on the previous reports mentioned above, the upregulation of *FGD5-AS1* consistently promotes cell proliferation, migration, and invasion and suppresses apoptosis. Our results indicated that KD of *FGD5-AS1* resulted in a significantly enhanced apoptosis in CCC-HEH-2 cells, which is consistent with these results. RNAseq analysis identified a substantial number of genes with differential expression resulted from the KD of *FGD5-AS1*, among which 354 genes were upregulated and 228 were downregulated. Since we performed a strict criterion in the differential expression analysis of RNAseq, *SMAD4*, *PTEN*, and *RBSN* were not identified as significantly changed, which is in contrast with our initial RT-qPCR validation of the *FGD5-AS1* KD effect on these mRNA targets. We further validated the expressions of *SMAD4* and *RBSN* using RT-qPCR in these samples and confirmed their significant downregulation in *FGD5-AS1* KD cells (Supplementary Figure 5). Both the upregulated and downregulated genes contained multiple known CHD genes (Figure 4D). Functional enrichment analysis indicated that only the upregulated genes were enriched in several biological processes such as blood vessel morphogenesis, as well as the CHD-related signaling pathways such as extracellular matrix assembly, Wnt, BMP, and ERK (Supplementary Table 5), and disease ontologies (e.g., cardiovascular disorders and non-cardiac disease), which indicates that *FGD5-AS1* might function in multiple systems. In contrast, the downregulated genes were not enriched in any functional items. Therefore, we suggested that the essential functional significance of *FGD5-AS1* in CHD should be its repression roles of gene expression in heart development. Consistently, we identified much more upregulated (increased accessibility) regions in the differentially accessible regions (376 had increased accessibility and 141 had reduced accessibility) through ATACseq. Based on gene-level region annotation for ATACseq, the intersection (10 genes) of the upregulated signals in RNAseq and ATACseq pointed out a possible regulatory route of *FGD5-AS1*. Our results implied an enrichment of known CHD genes (*VIT*, *HAS2*, and *ENPP2*) in the 10 genes. We did not observe any change in the peak signal around *FGD5-AS1* and its ceRNA targets (*SMAD4*, *PTEN*, and *NRAS*), which indicated that an epigenetic regulation mechanism was not involved in the ceRNA regulation of *FGD5-AS1*. Additionally, we also identified plentiful enriched known motifs of the transcription factors (e.g., *NKX6.1*, *RUNX1*, and *EN1*) within the differential accessible region sequences, which also highlighted the importance of the transcriptional regulation of *FGD5-AS1* besides the ceRNA mechanism. It is notable that *HAS2*, which encodes hyaluronan synthase 2, is responsible for hyaluronan production and mediation of the epithelium to mesenchyme. The disruption of *Has2* has been proven to abrogate normal cardiac morphogenesis (Camenisch et al., 2000). Considering that the GO enrichment results include multiple extracellular matrix-associated pathways (Supplementary Table 5) that are tightly associated with hyaluronan content, further exploration of such process is required to clarify the molecular basis of *FGD5-AS1* regulation in cardiovascular development. In the present study, we identified a lncRNA-driven ceRNA regulatory network in TOF heart tissues. The results reflected the gene expression regulatory features of the disease state in TOF heart tissues. However, there is still room to improve our results. There were no available data from healthy control samples in the gene expression network construction. If possible, further incorporation of differentially expressed genes generated from healthy *versus* disease samples would reveal the disease-specific ceRNA networks in CHD.

In summary, we identified the key lncRNA-driven ceRNA regulatory network in heart tissues with TOF. Through cell-based validation, we proved that the hub lncRNA *FGD5-AS1* not only functions as a ceRNA but also regulates the expression of its miRNA partner. Besides the ceRNA mechanism, we discovered that *FGD5-AS1* could also regulate multiple known CHD genes transcriptionally. Our study provided evidences of a candidate master regulator in cardiovascular development and CHD pathogenesis.

## Data Availability Statement

The datasets presented in this study can be found in online repositories. The names of the repository/repositories and accession number(s) can be found below: https://www.ncbi.nlm.nih.gov/geo/, GSE157626 and https://www.ncbi.nlm.nih.gov/geo/, GSE159464.

## Ethics Statement

The studies involving human participants were reviewed and approved by the Ethics Committee of the Shanghai Children’s Medical Center. Written informed consent to participate in this study was provided by the participants’ legal guardian/next of kin.

## Author Contributions

XYZ, YG, XPZ, XQZ, and YX performed the lab experiments. XYZ and BW performed the bioinformatics data analysis and wrote the manuscript. XYZ, QF, BW, and ZX contributed to writing and editing. All authors contributed to the article and approved the submitted version.

## Conflict of Interest

The authors declare that the research was conducted in the absence of any commercial or financial relationships that could be construed as a potential conflict of interest.

## Footnotes

1 https://www.encodeproject.org/

## Supplementary Material

Supplementary Figure 1 Four hub lncRNAs were identified in ceRNA regulatory network of TOF hearts. This network contains three types of genes, namely lncRNAs (upper left corner), mRNAs (upper right corner) and miRNAs (bottom).

Supplementary Figure 2 Validation of gene expression of *FGD5-AS1*, its target mRNAs and associated miRNAs in TOF tissues. qPCR was performed with 12 TOF patients right ventricular tissues for transcriptional expression of *FGD5-AS1* **(A)**, three target mRNAs **(B–D)**, and the four associated miRNAs **(E–H)**. Gene expression data are reported as 2^−ΔCt^ relative to the *GAPDH*. The correlation coefficients between the mRNAs and *FGD5-AS1* are shown in parentheses.

Supplementary Figure 3 Positive control for luciferase assays. The previous reported miRNA-mRNA target pair (hsa-miR-491/CRYAB) was validated in HEK 293 **(A)** and AC16 cell line, respectively **(B)**. n.s., not significant; The number of asterisks indicated the corresponding statistical significance (*p-value*). **p* < 0.05; ***p* < 0.01; ****p* < 0.001.

Supplementary Figure 4 Cell proliferation was measured by flow cytometry in *FGD5-AS1* KD CCC-HEH-2 cell line. n.s., not significant; The number of asterisks indicated the corresponding statistical significance (*p-value*). **p* < 0.05; ***p* < 0.01; ****p* < 0.001.

Supplementary Figure 5 *FGD5-AS1* decreased the transcriptional expression level of target mRNAs (*SMAD4 and RBSN*). **(A)** Initial RT-qPCR assays suggested decreased transcriptional level of two mRNAs in *FGD5-AS1* KD CCC-HEH-2 cell lines. **(B)** RT-qPCR assays confirmed the decreased expression of two mRNAs in *FGD5-AS1* KD CCC-HEH-2 samples analyzed by RNAseq. n.s., not significant; The number of asterisks indicated the corresponding statistical significance (*p-value*). **p* < 0.05; ***p* < 0.01; ****p* < 0.001.

Supplementary Table 1 Sequences for shRNA as well as miR mimics and primers for PCR; sequences of miRs-mRNAs/lncRNAs binding site for plasmids construction.

Supplementary Table 2 Clinical information of TOF patients.

Supplementary Table 3 Core lncRNA driven ceRNA network identified using gene expression data in 22 heart tissues with TOF.

Supplementary Table 4 Differential gene expression results of *FGD5-AS1* knockdown in CCC-HEH-2 cell line.

Supplementary Table 5 GO enrichment results of up regulated genes in *FGD5-AS1* KD CCC-HEH-2 cell line.

Supplementary Table 6 Differential accessible region analysis results of *FGD5-AS1* KD in CCC-HEH-2 cell lines.

Supplementary Table 7 Full lists of enriched known motifs of transcription factors within the differential accessible region sequences.
