# Supplementary figures and images for: FGD5-AS1 Is a Hub lncRNA ceRNA in Hearts With Tetralogy of Fallot Which Regulates Congenital Heart Disease Genes Transcriptionally and Epigenetically
Source: Front Cell Dev Biol. 2021 May 11;9:630634. doi: 10.3389/fcell.2021.630634 (PMC8144506; doi:10.3389/fcell.2021.630634)

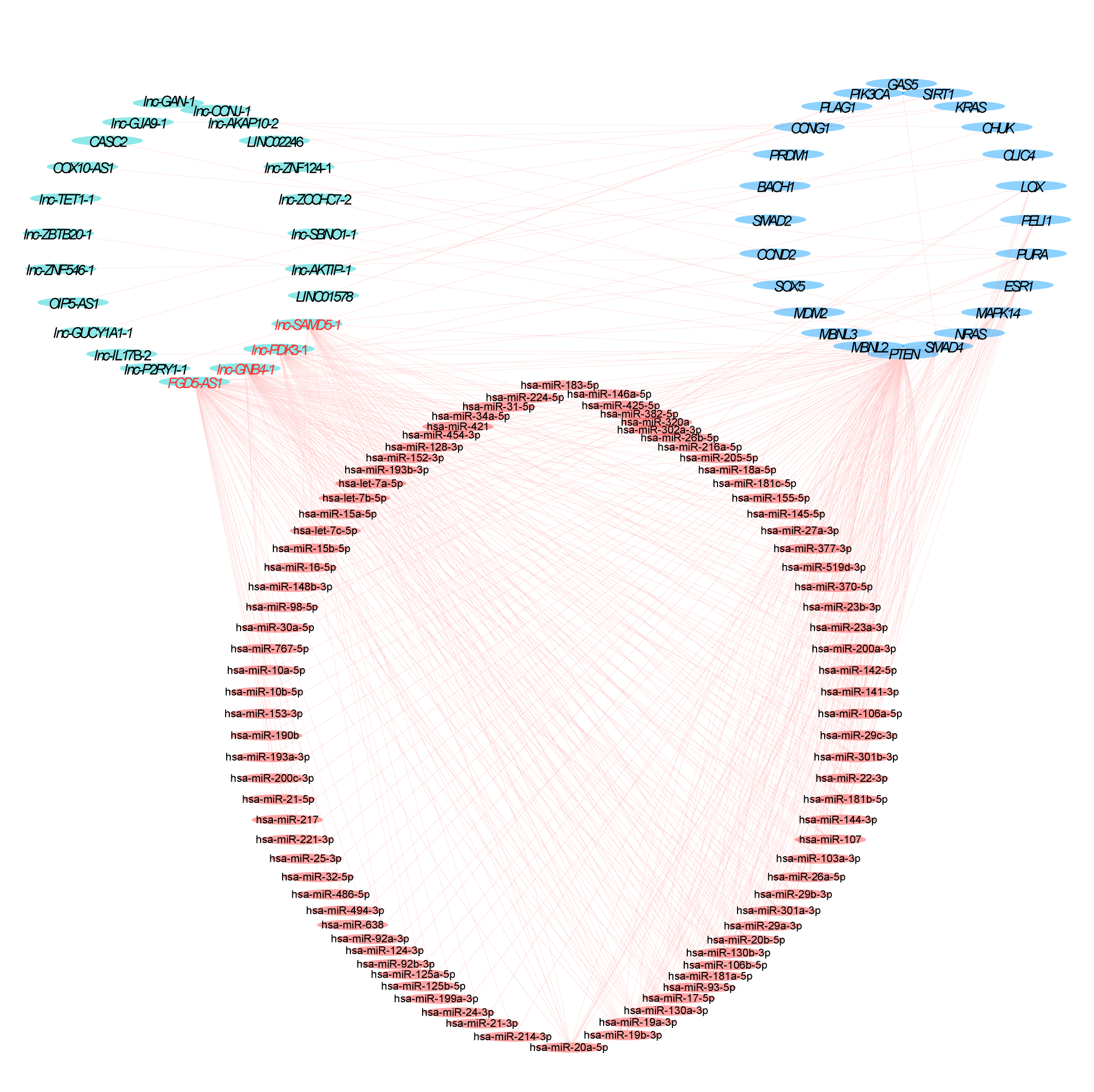

Supplement: Supplementary Figure 1 — Four hub lncRNAs were identified in ceRNA regulatory network of TOF hearts. This network contains three types of genes, namely lncRNAs (upper left corner), mRNAs (upper right corner) and miRNAs (bottom). [file Image_1.TIF]

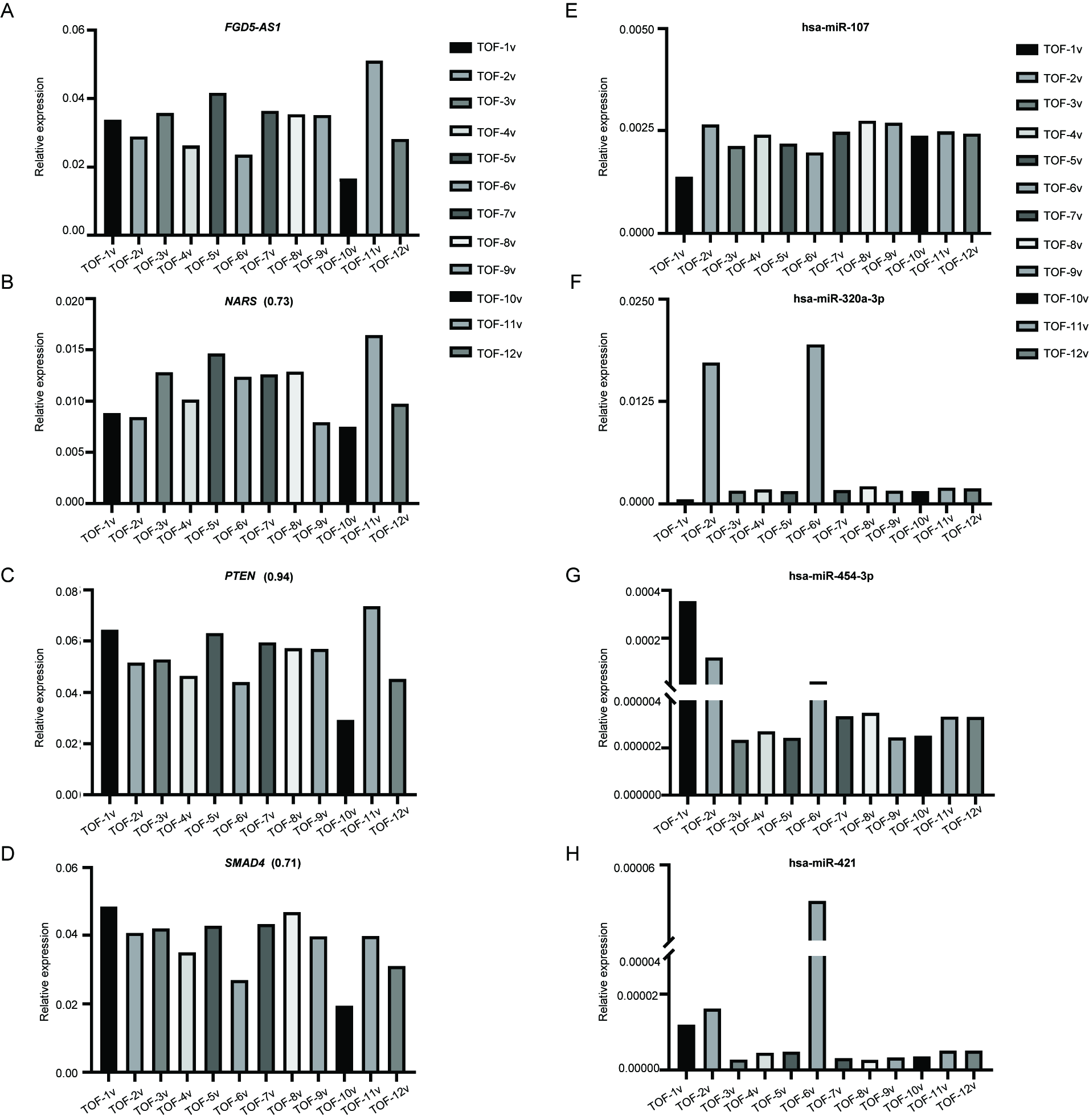

Supplement: Supplementary Figure 2 — Validation of gene expression of FGD5-AS1, its target mRNAs and associated miRNAs in TOF tissues. qPCR was performed with 12 TOF patients right ventricular tissues for transcriptional expression of FGD5-AS1 (A), three target mRNAs (B–D), and the four associated miRNAs (E–H). Gene expression data are reported as 2−ΔCt relative to the GAPDH. The correlation coefficients between the mRNAs and FGD5-AS1 are shown in parentheses. [file Image_2.TIF]

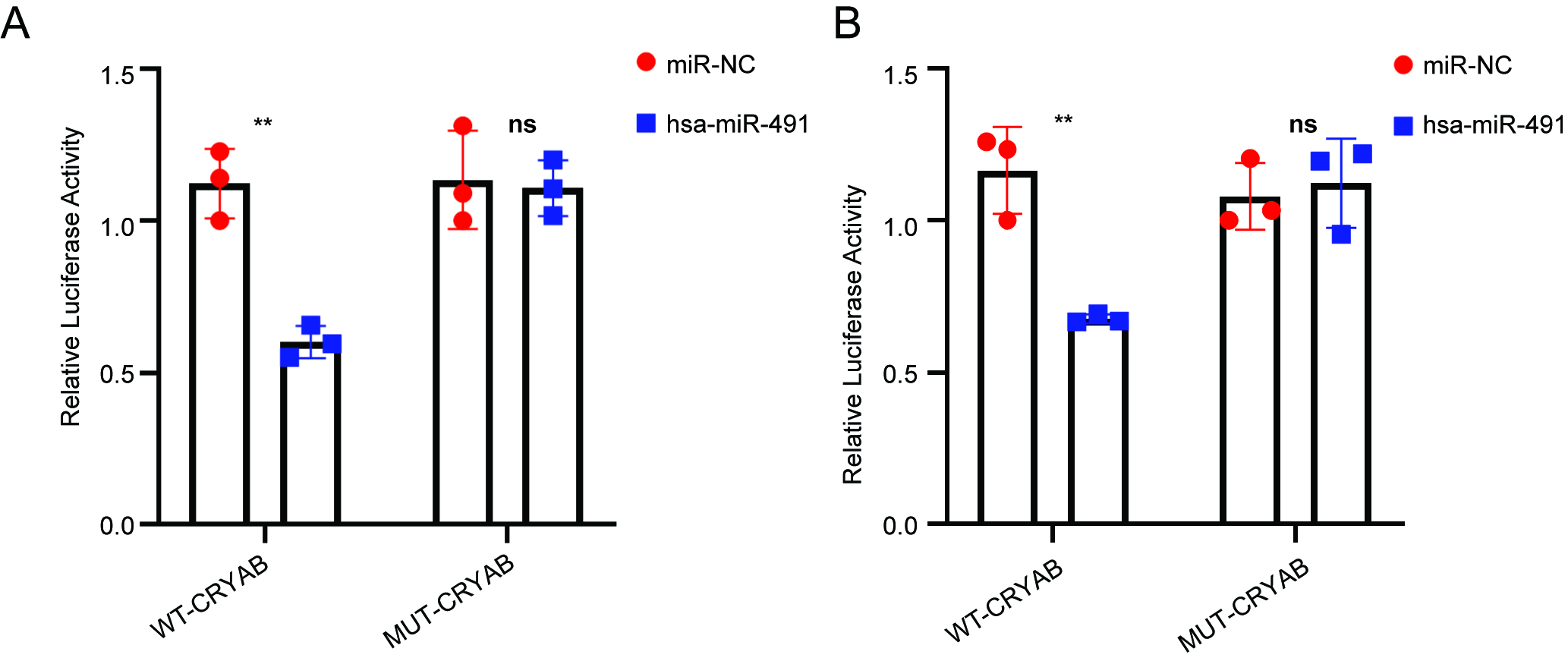

Supplement: Supplementary Figure 3 — Positive control for luciferase assays. The previous reported miRNA-mRNA target pair (hsa-miR-491/CRYAB) was validated in HEK 293 (A) and AC16 cell line, respectively (B). n.s., not significant; The number of asterisks indicated the corresponding statistical significance (p-value). *p < 0.05; **p < 0.01; ***p < 0.001. [file Image_3.TIF]

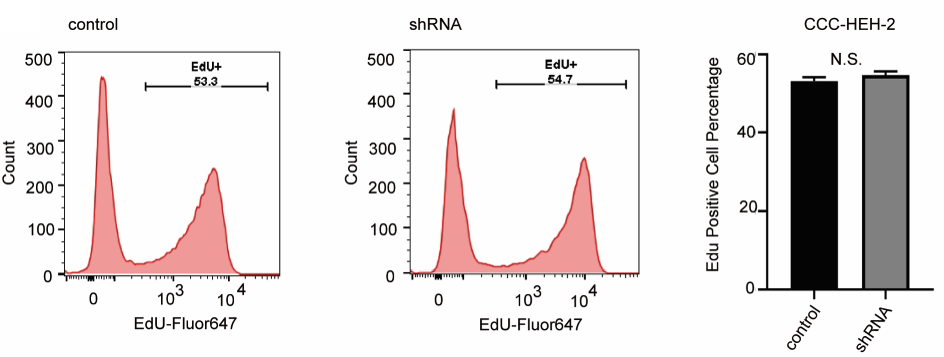

Supplement: Supplementary Figure 4 — Cell proliferation was measured by flow cytometry in FGD5-AS1 KD CCC-HEH-2 cell line. n.s., not significant; The number of asterisks indicated the corresponding statistical significance (p-value). *p < 0.05; **p < 0.01; ***p < 0.001. [file Image_4.tif]

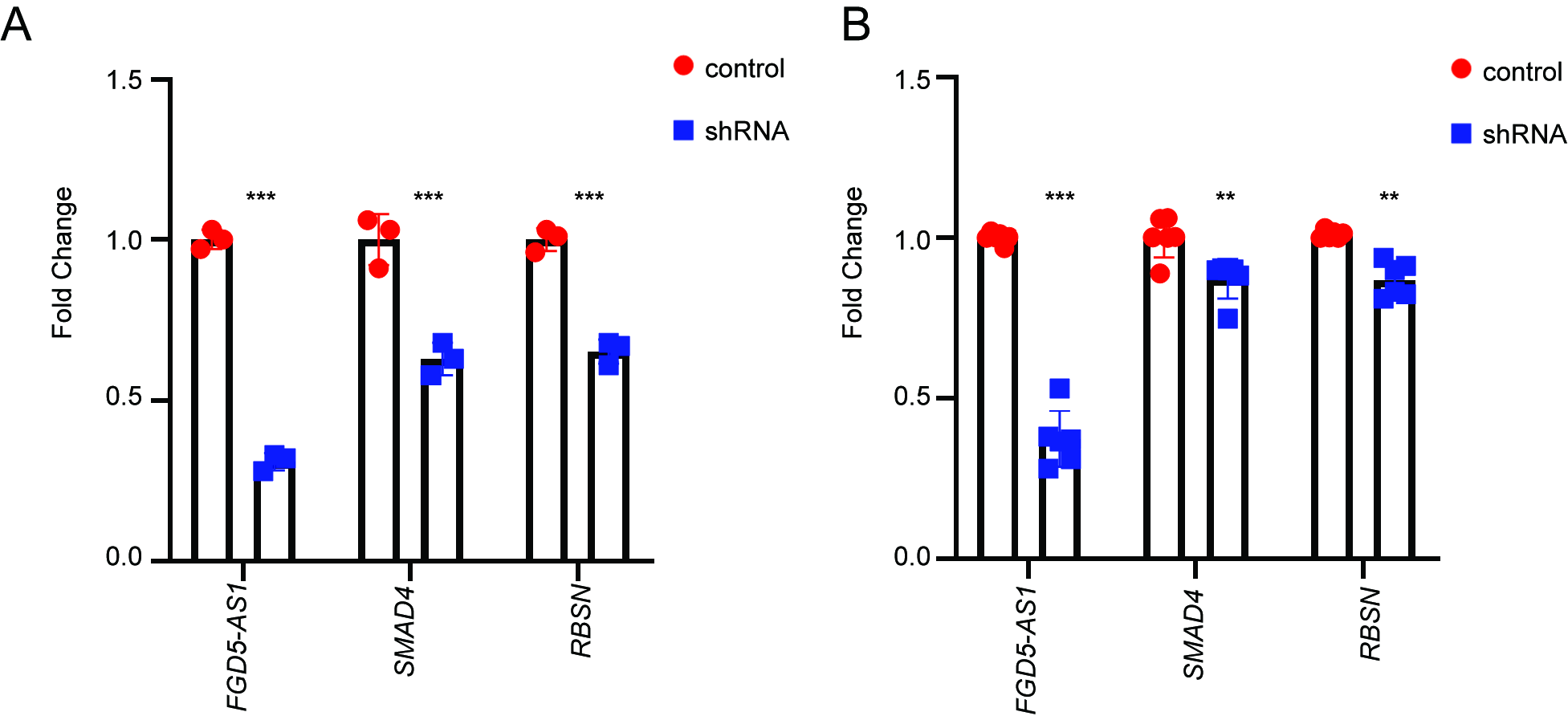

Supplement: Supplementary Figure 5 — FGD5-AS1 decreased the transcriptional expression level of target mRNAs (SMAD4 and RBSN). (A) Initial RT-qPCR assays suggested decreased transcriptional level of two mRNAs in FGD5-AS1 KD CCC-HEH-2 cell lines. (B) RT-qPCR assays confirmed the decreased expression of two mRNAs in FGD5-AS1 KD CCC-HEH-2 samples analyzed by RNAseq. n.s., not significant; The number of asterisks indicated the corresponding statistical significance (p-value). *p < 0.05; **p < 0.01; ***p < 0.001. [file Image_5.TIF]
